# The Food Energy/Protein Ratio Regulates the Rat Urea Cycle but Not Total Nitrogen Losses

**DOI:** 10.3390/nu11020316

**Published:** 2019-02-01

**Authors:** Laia Oliva, Marià Alemany, Xavier Remesar, José-Antonio Fernández-López

**Affiliations:** 1 Department of Biochemistry and Molecular Biomedicine, University of Barcelona, Faculty of Biology, 08028 Barcelona, Spain; laia.oliva@ub.edu (L.O.); malemany@ub.edu (M.A.), xremesar@ub.edu (X.R.); 2 Institute of Biomedicine, University of Barcelona, 08028 Barcelona, Spain; 3 Centro de Investigación Biomédica en Red Fisiopatología de la Obesidad y Nutrición (CIBER OBN), 08028 Barcelona, Spain

**Keywords:** urea cycle, nitrogen balance, protein/energy ratio, rat

## Abstract

Nitrogen balance studies have shown that a portion of the N ingested but not excreted is not accounted for. We compared several diets (standard, high-fat, high-protein, and self-selected cafeteria) to determine how diet-dependent energy sources affect nitrogen handling, i.e., the liver urea cycle. Diet components and rat homogenates were used for nitrogen, lipid, and energy analyses. Plasma urea and individual amino acids, as well as liver urea cycle enzyme activities, were determined. Despite ample differences in N intake, circulating amino acids remained practically unchanged in contrast to marked changes in plasma urea. The finding of significant correlations between circulating urea and arginine-succinate synthase and lyase activities supported their regulatory role of urea synthesis, the main N excretion pathway. The cycle operation also correlated with the food protein/energy ratio, in contraposition to total nitrogen losses and estimated balance essentially independent of dietary energy load. The different regulation mechanisms observed have potentially important nutritional consequences, hinting at nitrogen disposal mechanisms able to eliminate excess nitrogen under conditions of high availability of both energy and proteins. Their operation reduces urea synthesis to allow for a safe (albeit unknown) mechanism of N/energy excess accommodation.

## 1. Introduction

Dietary excess of amino acid N, an obvious consequence of high-protein diets, necessarily induces its oxidation for energy, thus increasing urea synthesis [1]. This function is mainly carried out by the liver, which plays an active role in the adaptation of whole-body nitrogen homoeostasis to dietary protein, possibly via glucagon [2]. The control role of liver on the disposal of ammonium-N and excess amino-N is a critical process for the maintenance of body–N homeostasis. Liver urea production is both a tight control system to prevent the loss of valuable amino acid N but also the best-controlled outlet for excretion of excess N. This way, daily dietary variations in amino acid availability are counteracted in significant part through adjustments in liver metabolic function. This mechanism, used to maintain N homeostasis, is complemented by other tissues and pathways, which are in part practically unknown.

A quantitative analysis of nitrogen balances has shown that a significant portion of the N ingested but not excreted as urea (or other N-containing catabolites) is not accounted for [3]. It has been postulated that the differences may be justified (at least in part) by respiratory loss of nitric oxide [4], or even by the direct release of nitrogen gas [5], but so far no definitive explanation has been found for this “nitrogen gap”. Although this deficit has been observed with different diet types, its extent is higher when using high-energy cafeteria diets [6]. Cafeteria diets are made up of palatable foods in which the range and variety of offered food taste, energy content, and texture induces a marked hedonic-driven increase in food consumption [7,8]. Contrary to high-protein diets, cafeteria diets decrease the operation of the liver urea cycle. The consequence is a lower overall urinary excretion of N [9], not paralleled by a compensatory increase in protein accrual [10], in spite of maintained or increased protein intake.

In this study, we compared several diets with well-established differences in their content of protein, lipids, and overall energy. In addition to a standard diet (the usual rat chow), we used a cafeteria diet and a high-fat diet matched in composition to the standard rat chow but supplemented with oil rich in saturated fat, which has a moderate obesogenic capacity [11,12]. The fat content of the high-fat diet (ca. 40%) was selected to coincide with the percentage of fat self-selected by rats using our simplified cafeteria diet model [13,14]. Finally, we used an isoenergetic, high-protein diet model also matched in composition (except for protein) to the standard rat chow.

The match in nutrients, except lipids or protein, allowed us to establish comparisons based only on these aspects of diet, limiting possible interference by other dietary components [15]. By using this array of partially superimposable diet comparisons, we intended to analyze the paradox of decreased urea synthesis of rats fed a cafeteria diet and also determine how diet energy may affect nitrogen partition, including hepatic operation and overall efficiency of the urea cycle.

## 2. Materials and Methods

### 2.1. Diets

Table 1 presents the composition of the diets used. The standard diet (Teklad 2014, Teklad diets, Madison, WI, USA) contained 20% of digestible energy derived from proteins, 13% from lipids, and 67% from carbohydrates (including 0.10% from oligosaccharides). This diet contained essentially plant-derived aliments.

The high-fat diet was prepared by the addition of coconut oil (Escuder, Rubí, Spain) to the standard chow coarsely ground. The initial mixture contained 33 parts (by weight) of standard chow, 4 of coconut oil, and 16 of water (added to favor the mixture and kneading of the paste). The high-protein diet was prepared in a similar way, although in this case the mix contained 16.5 parts (by weight) of standard chow, 2.35 of casein, 2.05 of fish gelatin, 0.20 of sunflower oil, and 17 parts of water. In both cases, the “dough” was thoroughly kneaded to form a rough paste that was extruded using cut-end syringes to form 1 × 6 cm cylindrical pellets, which were dried at 40 °C until achieving the required consistency [15]. Aversion tests to these diets gave negative results, i.e., they were not different from the standard diet.

The simplified cafeteria diet was formed by excess offering of the standard chow pellets, plain cookies spread with liver pâté, bacon, water, and milk supplemented with 300 g/L sucrose and 30 g/L of a mineral and vitamin supplement (Meritene©, Nestlé, Esplugues de Llobregat, Spain) [13,14]. All components were kept fresh (i.e., renewed daily). From the analysis of the ingested items and diet composition, we calculated that approximately 40% of ingested energy was derived from lipids, 12% from protein, and 47% from carbohydrates (23% from oligosaccharides and 24% starch).

### 2.2. Animals and Experimental Setup

All animal handling procedures and the experimental setup were devised and carried out in accordance with the animal handling guidelines of the European, Spanish, and Catalan authorities. The Committee on Animal Experimentation of the University of Barcelona authorized the specific procedures used (# DAAM 6911). The assumedly excessive suffering of the animals when placed in metabolic cages, due to their necessary isolation, formally prevented the direct measurement of the loss of nitrogen in urine and feces along the period studied. Since previous published data from our group [3,16,17] using animals fed a standard, cafeteria, or high-protein diet, when combined, showed the existence of a close linear correlation between circulating urea levels and global nitrogen elimination (*r* = 0.9621), plasma urea concentration was used as an indirect indicator of overall nitrogen loss in urine and feces.

Ten-week-old male and female Wistar rats (Janvier, Le Genest Saint-Isle, France) were used (*n* = 52). The animals were randomly divided into four groups (*n* = 6–8 for each sex) and were fed ad libitum either the standard, high-fat, high-protein, or cafeteria diets for 30 days. All animals had free access to water. The rats were housed (in same-sex pairs) in solid-bottom cages with wood shards as bedding material and were kept in a controlled environment (lights on from 08:00 to 20:00, temperature 21.5–22.5 °C, and 50%–60% humidity). Body weight and food consumption were recorded daily. The calculation of ingested food in cafeteria diet-fed rats was done as previously described by weighing the differences in food offered and debris left (corrected for desiccation) [8]. 

On day 30, at the beginning of the light cycle, the rats were anesthetized with isoflurane and then killed by exsanguination through the exposed aorta using a dry-heparinized syringe. Plasma was obtained by centrifugation and kept at −20 °C until processed. The liver was dissected, weighed, and a liver sample was frozen with liquid nitrogen and maintained at −80 °C until processed. The content of the gastrointestinal tract was cleaned, and the carcass (and remaining blood, liver, and debris) was sealed in polyethylene bags, which were subsequently autoclaved at 120 °C for 2 h [6]: The bag contents were weighed and then minced to a smooth paste with a blender, thus obtaining a “total rat” homogenate. The constituents of the diets given to the animals were also ground and homogenized, and then subjected to the same analytical procedures.

### 2.3. Analytical Procedures

Nitrogen content was measured with a semiautomatic Kjeldahl procedure using a ProNitro S system (JP Selecta, Abrera, Spain). Initial rat nitrogen content was estimated from the percentage of N previously published (at day 0) of control animals from the same stock (and provider), age, and sex [18,19], and was adjusted using the initial body weight of each animal used in this experiment. Total accrued N was calculated from the values of body nitrogen obtained at the end of the experiment and discounting the estimated initial nitrogen content. The conversion of rat N content to rat protein content was done using the specific 5.5 conversion factor for whole rat protein content previously measured experimentally by us [20].

Carcass and food lipid content were measured by weight using a classical solvent homogenization-extraction method with trichloromethane/methanol 2:1 (*v*/*v*) [21]. Water body content was measured from carcass samples by differential weighing, before and after 24 h at 110 °C. The energy content of diet components was determined using a bomb calorimeter (C7000, Ika, Staufen, Germany). Energy intake was calculated from daily food consumption converted with the energy equivalence of the different foods and components measured with the bomb calorimeter.

Plasma urea was measured with kit #11537 (Biosystems, Barcelona, Spain). Individual amino acids were analyzed with a Biochrom 30 (Biochrom Ltd., Cambridge, UK) amino acid analyzer, using plasma samples deproteinized with 100 g/L of trifluoroacetic acid.

### 2.4. Enzyme Activity Analyses

Frozen liver samples were homogenized in buffer using a tissue disruptor (Ultraturrax IKA-T10, Ika, Russia) at 2–4°C. Homogenates for carbamoyl-P synthase and ornithine carbamoyl-transferase activity measurement were prepared using 10 volumes of chilled 70-mM hepes buffer pH 7.4 containing 1 mM dithiothreitol, 50 mM KCl, 1 g/L Triton X-100, and 1 g/L lipid-free bovine serum albumin (all from Sigma-Aldrich, St Louis, MO, USA). Homogenates for the analyses of the other enzymes were prepared with 10 volumes of chilled Krebs-Ringer bicarbonate buffer pH 7.4 containing 1 g/L Triton X-100, 1 mM dithiothreitol, and 1 g/L lipid-free bovine serum albumin. The homogenates were coarsely filtered through a nylon hose to eliminate large debris. They were kept on ice and used for enzyme activity analyses within 2 h. Tissue protein content was estimated with the Lowry method [22], using the corresponding homogenization buffer (i.e., containing albumin) as a blank. Enzyme activities were expressed per unit of protein weight and total liver content. 

Carbamoyl-P synthase activity was estimated from the incorporation of ^14^C-bicarbonate into carbamoyl-P, and was converted to hydroxyurea [23]. Diluted (1:4 v/v) homogenates (50 µL) were mixed with a reaction buffer containing ATP-Na_2_, N-acetyl glutamate, and magnesium acetate (all from Sigma-Aldrich): The final concentrations were 20 mM, 5 mM, and 20 mM, respectively. The reaction was started with 50 µL of ammonium bicarbonate and a sodium-^14^C-bicarbonate (PerkinElmer, Bad Neuheim, Germany) mixture (final concentration: 50 mM, 5 kBq/tube), and was carried out at 37 °C for 0, 8, or 16 min. The reaction was stopped by introducing 200 µL of the reaction mixture into tubes kept on ice containing 30 µL of 2-M hydroxylamine-HCl and was rapidly put at 95 °C until total evaporation. All labeled carbamoyl-P formed was converted to labeled hydroxyurea and remained in the tube. The whole-tube contents were counted.

Ornithine carbamoyl transferase activity was measured from the reaction of condensation of carbamoyl-P and ornithine to yield citrulline (adapted from [24]). Aliquots (40 µL) of diluted homogenates (1:49 v/v) were mixed with urease S (Boehringer Mannheim, Mannheim, Germany) and a reaction buffer containing hepes, KCl, MgCl_2_, and ornithine (all from Sigma-Aldrich): The final concentrations were, respectively, 100 µkat/L, 50 mM, 33 mM, 4.5 mM, and 10 mM. The reaction was started by adding 15 µL of lithium carbamoyl-P (Sigma-Aldrich) (final concentration 6.7 mM) and was carried out at 37 °C for 0, 5, or 10 min. The reaction was stopped by introducing 200 µL of the reaction mixture into chilled tubes containing 600 µL of reaction buffer: Diacetyl monoxime (59.3 mM), antipyrine (7.2 mM), and FeCl_3_ (0.3 mM) diluted with 3.75% acetic acid (v/v) and 30% H_2_SO_4_, prepared as previously described [24] (all products were from Sigma-Aldrich). The color reaction was developed at 100 °C for 30 min in a boiling water bath. Absorbances (including standards and blanks) were measured at 450 nm using a plate reader spectrophotometer (ELx808 Ultra Microplate Reader, Biotek, Winooski, VT, USA).

The methods used for arginino-succinate synthase and lyase and arginase enzyme activities have been previously described in detail [25].

### 2.5. Statistical Procedures

Statistical comparisons were done using two-way ANOVA analyses (factors: Sex and diet) and the post hoc Bonferroni test, using the Prism 5.0 program (GraphPad Software Inc, La Jolla, CA, USA). Differences were considered significant when the *p*-value was <0.05.

## 3. Results

### 3.1. Body Balance and Nutrients Intake

Table 2 shows body weight, body composition, and energy, as well as macronutrients intake, in all groups during the 30-day study. As expected, males showed a higher food intake, which also induced higher energy and macronutrient intake than in females. When diets were compared, rats fed the cafeteria diet had higher values for both weight gain and energy, carbohydrates, and lipid consumption. Cafeteria diet-fed rats showed a higher percentage of body fat. The rise in lipid content was compensated by lower percentages (but not in absolute values) of water and protein compared to the other diet groups. Again, as expected, the high-fat diet group showed the second-highest lipid intake, while the high-protein diet group had the highest protein intake. The freedom to select food items according to the individual whims of the rats fed the cafeteria diet resulted in only slight variations in the protein/energy consumption ratios between different cafeteria-fed animals (showing no significant differences between sexes: 11.3% ± 0.4% for females and 12.3% ± 0.5% for males), as depicted in Table 1. In all the other groups, there could not be such variation because the diet composition was fixed.

### 3.2. Nitrogen Balance

Table 3 shows the nitrogen balance of the different groups of animals. The N content at the end of the experiment among the rats fed with different diets did not show significant differences in spite of wide differences for ingested nitrogen. Nevertheless, the males showed higher N content than females due to their larger size. Since the amount of accrued N was only a small proportion of the total N ingested, both ingested N and N losses presented similar profiles: Higher values in males, but showing marked differences between groups. The highest intakes and losses of nitrogen corresponded to the high-protein diet, followed by the cafeteria diet, with the high-fat diet showing the lowest values. The groups fed the standard and cafeteria diets had the highest values for protein accrual.

### 3.3. Plasma Metabolites

Table 4 shows the plasma amino acid levels in the four diet groups studied at the end of the experiment. Several amino acids did not show significant concentration changes (Asp, Thr, Arg, citrulline, Met, Trp, Tyr), although in others there were differences related to sex, with higher values in males (Asn, Glu, Ala, His, Phe, Gly, ornithine): The only amino acid with higher plasma levels in females was Lys. When comparing the effects of diets, the high-protein group showed higher plasma levels (Gly, Pro, Val, Leu, Ile, Lys, ornithine, and Tyr). In the high-fat diet group, only higher Gln and Tyr levels were observed.

**Figure 1:**
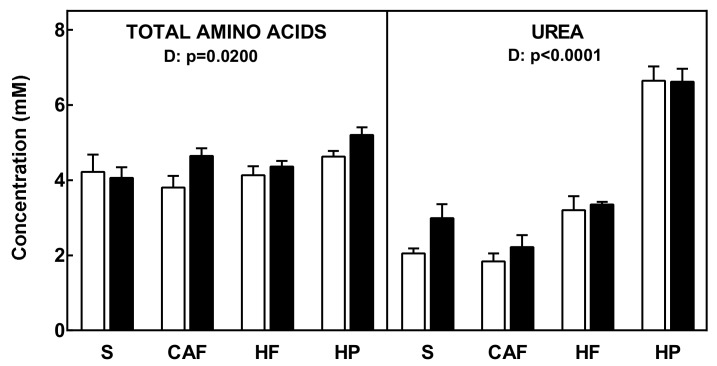
Plasma total amino acids and urea levels of female and male rats fed a standard, cafeteria, high-fat, or high-protein diet for 30 days. The data correspond to the mean ± SEM of 6–8 different animals. Females are represented by white bars, and males by black. Abbreviations: S is standard diet, CAF is cafeteria diet, HF is high-fat diet, and HP is high-protein diet. Statistical analysis was done using a two-way ANOVA program for diet (D) and sex (S). Only significant values are represented.

The concentrations of total amino acids and plasma urea are shown in Figure 1. Changes between groups for total amino acid levels were minimal, with the differences reflecting the data presented for individual amino acids, i.e., slightly higher overall concentrations in the high-protein diet group. However, plasma urea levels changed much more widely, with higher values for the high-protein diet and the lowest for cafeteria-fed animals.

### 3.4. Enzyme Activities

**Figure 2:**
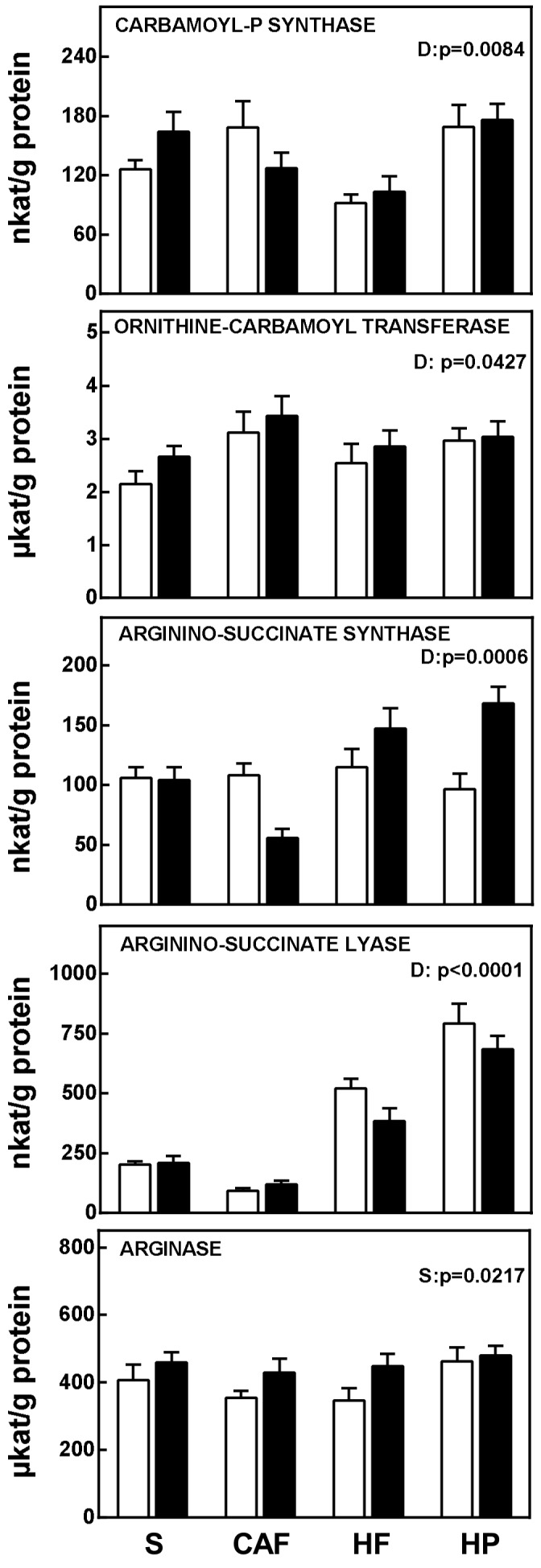
Enzyme activities related to the urea cycle in the liver of male and female rats fed a standard, cafeteria, high-fat, or high-protein diet for 30 days. The data correspond to the mean ± SEM of 6–8 different animals, and are all expressed per gram of tissue protein. Females are represented in white bars, and males in black. Abbreviations: S is standard diet, CAF is cafeteria diet, HF is high-fat diet, and HP is high-protein diet. Statistical analysis was done using a two-way ANOVA program for diet (D) and sex (S). Only significant values are represented.

Figure 2 shows liver urea cycle-related enzyme activities. All enzyme activities (except arginase) were affected by diet. However, while the lowest values, in the case of carbamoyl-P synthase, were observed in the high-fat diet group, in the case of arginine-succinate synthase (especially in males) and lyase, the lowest activities were those of the cafeteria diet-fed rats. In general, high protein intakes were paralleled also by higher urea cycle enzymatic activities. The only enzymatic activity significantly affected by sex was arginase activity, with lower values in females in both the cafeteria and high-fat diet groups.

### 3.5. Correlations

**Figure 3:**
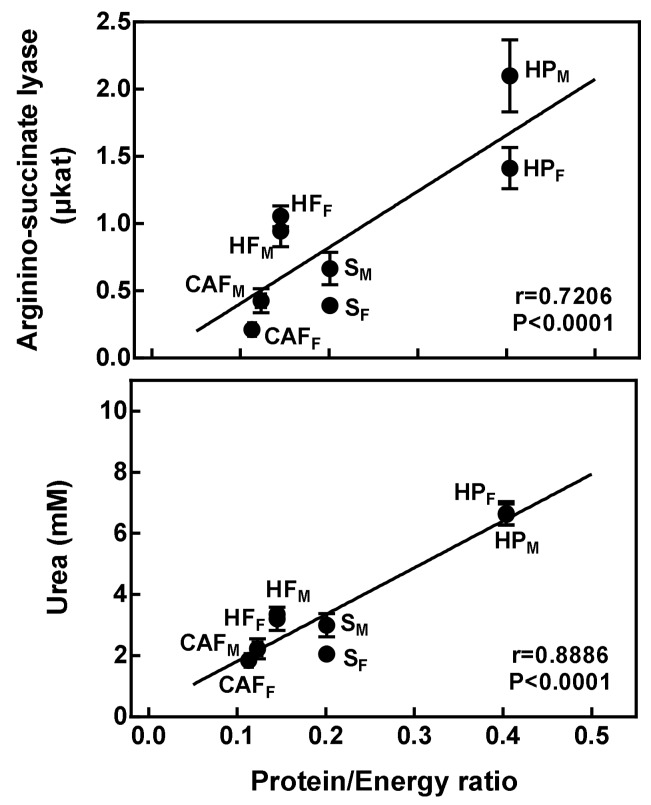
Relationship between the diet protein/energy ratio with total liver arginine-succinate lyase activity and plasma urea levels. The protein/energy ratio refers to the energy ingested derived from proteins versus total energy ingested. Lines of regression, correlations, and *p*-values were calculated using all individual data (*n* = 52). Abbreviations: S is standard diet, CAF is cafeteria diet, HF is high-fat diet, HP is high-protein diet, F means female rats, and M means male rats.

Total liver activities (calculated as a measure of global catalytic liver capacity) of arginino-succinate synthase and lyase were significantly correlated with plasma urea levels: Correlation values were *r* = 0.322 for arginino-succinate synthase (*p* = 0.0200) and *r* = 0.762 for arginino-succinate lyase (*p* < 0.0001). Arginino-succinate lyase activity and plasma urea levels were correlated with the diet protein/energy ratio (Figure 3).

## 4. Discussion

We studied the effects of diets with different contents of energy, lipids, and protein, but which were essentially uniform in most of their other components. Using this approach, we were able to show the importance of the food protein/energy ratio in the control of urea cycle operation, in contraposition to total nitrogen losses and nitrogen balance, which were essentially independent of the diet energy content. The main limitation of the study was the impossibility of obtaining direct measures of nitrogen losses in urine and feces along the period studied: To overcome this limitation, we used an indirect measure of total N excretion, plasma urea levels.

The markedly different regulation observed between nitrogen losses and urea cycle operation was apparent by the lack of direct relationship between urea synthesis and obvious excess N, with energy playing a key role in the mode of disposal of excess dietary N. These differences have potentially important consequences, since they support the existence of an additional nitrogen disposal pathway able to eliminate excess nitrogen under situations of high availability of both energy and proteins. The urea cycle—in fact, all of the N disposal mechanisms for noncarnivore mammals—is centered in a tight control of N losses, which becomes a problem in the “rare” cases when both energy and N are in excess. The existence of an alternative pathway overcomes this question, with the apparent contradictory decrease in the operation of the urea cycle, which can be assumed to be a direct correlate of body–N homeostasis.

The quantitative importance of such an additional mechanism is considerable and grants the need for further study of its consequences in the maintenance of body–nitrogen balance under normal conditions and under pathological situations such as metabolic syndrome.

Homoeostatic maintenance of glycaemia and the preservation of body protein via the supply of amino acids (both general amino-N and essential ones) are critical for survival [26]. In our experimental model, the animals had no problems with amino acid availability in any of the diets tested. In fact, final body N was maintained and was unaffected regardless of diet: The only plausible question related to amino acids was the need to dispose of their possible excess. Neither was there any deficit in dietary energy availability, since the digestible food energy density of the cafeteria and high-protein diets was similar to that of the standard diet, and that of the high-fat diet was even higher: The main differences between diets rested in the proportion of nutrients and the relationships between them and with total energy intake.

The fact that there was no energy deficit in these animals was confirmed by the weight gain of animals fed the high-fat and high-protein diets, similar to that of control animals. These results agreed with data previously described [12,27], although in both cases (higher or normal body weight gain for the high-fat diet and lower or normal body weight gain for the high-protein diet), the magnitude of the changes depended on the particular diet composition used [12,27]. 

The known obesogenic effects of cafeteria diets were confirmed by a significant increase in body weight [18], more marked in males and largely caused by the accumulation of fat, mainly in adipose tissue, although an increase in fat content affects all tissues [13]. This increase is accompanied by parallel, but less extensive, increases in lean body mass [28], with enhanced protein deposition (in absolute terms) [29]. This is in part driven by higher amino-N availability, paralleled by lower urea N excretion [9].

Despite the important differences in N intake, circulating amino acids showed only minor differences between groups, remaining practically unchanged, especially when compared to the marked variability of plasma urea. Food intake increases plasma amino acids, which stimulates insulin release and mTOR-dependent protein synthesis in muscle [1]. Most excess amino acids are oxidized (especially the non-essential or the highly available essential ones) [1,30]. These mechanisms are well regulated, resulting in remarkably maintained plasma amino acid levels even under conditions of protein restriction [31]. The liver is, at least in part, responsible for the limited effect of nutrient intake on plasma amino acid levels, since it disposes of most of the excess amino acids provided by a normal diet [31]. However, the extent to which amino acids are used by the liver is different for alanine and glutamine [32], which act as inter-organ vectors carrying amino-N and ammonium to the liver essentially for disposal [33], than for branched-chain amino acids, most of which are oxidized elsewhere [34]. 

Estimated enzyme activities are not direct approximations of in vivo enzyme function, but are generally taken as a correlate of the total amount of functional enzyme (i.e., that corresponding to *V_max_*) and, consequently, of overall enzyme ability to carry out its physiological function. Since this study was done using different diet conditions on rats of both sexes, the finding of significant correlations between plasma urea concentrations and arginine-succinate synthase and lyase activities reinforce their assumedly important regulatory role in the control of liver urea cycle operation [35,36]. This correlation, akin to control of hepatic urea production, also suggests that circulating urea may be considered a fair index of urea cycle operation. This is strengthened by the fact that plasma urea levels vary in parallel to urinary losses: High plasma urea levels parallel high urine urea excretion following a high-protein diet [37,38,39], and both lower plasma urea and limited urinary losses have been found in rats fed cafeteria diets [6,9,39,40]. 

Increased protein intake, obvious in high-protein diets, initiates at least two major metabolic responses: An increase in protein synthesis (largely in muscle) driven by insulin [41,42] paralleled by an increased hepatic production of urea [43]. The urea cycle operates in part to prevent porta or hepatic metabolism-derived ammonium from entering systemic circulation [44]. However, a cafeteria diet, also characterized by a higher intake of protein-derived amino acids, resulted in both low plasma urea and excretion. As far as we know, the diet fat content did not affect per se the functionality of the urea cycle. This is a direct conclusion of the present study, and also confirms previous reports [25], since although the cafeteria diet lowered urea cycle operation, the high-fat diet did not.

It can be assumed that the relatively low contribution of protein-derived energy in relation to the total energy budget of the cafeteria diet could induce strongly unwanted protein-sparing mechanisms similar to those observed in situations of amino acid scarcity: Starvation [45] or diluted diets [46]. In all these cases, amino acid oxidation is diminished with a parallel decrease in urea synthesis [47], but circulating amino acids are maintained by the well-balanced equilibrium between protein synthesis and proteolysis [48]. There is a conflict between the setting of amino acid sparing mechanisms (lower urea production, higher intestinal absorption, decreased urinary N excretion, etc. [49]) and their higher availability derived from the ingestion of roughly the same amount of amino-N and the effectiveness of the amino-N sparing mechanisms. Thus, the only outlets available for disposal of the excess amino-N are a shift to protein turnover favoring higher protein accrual and the unexplained and not yet understood mechanisms that have been defined as a “nitrogen gap” [3,6] and that have been described as a direct production of nitrogen gas [5]. It has been postulated that this “nitrogen gap” excretion process is related to the metabolism of arginine [39] and is influenced by sex [25]. It may be speculatively suggested that a lower operation of the guanidine-handling enzymes of the urea cycle in the liver of cafeteria diet-fed rats may allow an increased derivation of intermediates toward this so far unknown path for N excretion.

The factors that regulate the fate of N, described here, may also be important in humans since, western societies are characterized by an excessive intake of both protein and energy. The excess of protein causes an overload of N, whose elimination is hindered by the consequent excess of energy. The main question is how metabolic machinery can override the strong protective mechanisms preventing N-wasting under conditions of excess energy [50]. It is possible to venture that not only the excess of nutrients, but also the imbalance in the proportion of nutrients, can have important metabolic consequences: Part of the dysregulation found in the metabolic syndrome may be an unwanted consequence of this N disposal conflict.

## 5. Conclusions

In conclusion, the food protein/energy ratio has potentially important consequences in the control of urea cycle operation, since high-energy diets tend to inhibit urea cycle function: The different regulation observed hints at the existence of an additional nitrogen disposal pathway capable of eliminating excess nitrogen in situations of high availability of both energy and proteins.

## Figures and Tables

**Table 1:** Diet energy and macronutrients content.

	Standard Diet	Cafeteria Diet *	High-Fat Diet	High-Protein Diet
Crude energy (kJ/g)	16.5	12.4 ± 0.2	18.8	17.4
Digestible energy (kJ/g)	12.1	12.0 ± 0.1	14.6	12.4
Theoretical energy derived from (%): Carbohydrates Protein Lipids	67.0 20.1 13.0	47.3 ± 1.2 11.9 ± 0.3 40.3 ± 0.6	48.7 14.5 36.8	47.7 40.4 11.6

* The data for the rats fed the cafeteria diet were the mean ± SEM of six pairs of rats; no significant differences between sexes were observed.

**Table 2:** Body weight increase and composition, energy, and macronutrients intake.

	Standard Diet		Cafeteria Diet		High-Fat Diet		High-Protein Diet	
	Female	Male	Female	Male	Female	Male	Female	Male
Weight increase (g)	39.5 ± 4.3 ^A^	79.2 ± 8.2 ^a^	73.6 ± 6.9 ^B^	126 ± 3 ^b^	27.5 ± 1.7 ^A^	82.8 ± 6.3 ^a^	27.7 ± 3.8 ^A^	68.7 ± 1.5 ^a^
Body composition (%) Water Lipids Proteins	59.6 ± 1.1 ^A^ 17.3 ± 0.1 ^A^ 18.6 ± 0.1 ^A^	61.2 ± 0.6 ^a^ 15.7 ± 1.0 ^a^ 19.6 ± 0.7 ^a^	54.1 ± 1.5 ^B^ 22.1 ± 1.7 ^B^ 16.2 ± 0.2 ^B^	59.0 ± 0.5 ^a^ 17.5 ± 0.9 ^a^ 17.1 ± 0.5 ^b^	61.2 ± 0.37 ^A^ 14.9 ± 0.7 ^AC^ 18.2 ± 0.2 ^A^	61.3 ± 0.4 ^a^ 15.9 ± 0.5 ^a^ 16.8 ± 0.1 ^b^	61.2 ± 0.6 ^A^ 13.1 ± 0.6 ^C^ 18.3 ± 0.1 ^A^	61.4 ± 0.7 ^a^ 12.7 ± 0.5 ^b^ 18.7 ± 0.3 ^a^
Intake (MJ) Energy Carbohydrates Lipids Proteins	6.33 ± 0.35 ^A^ 4.24 ± 0.17 ^A^ 0.79 ± 0.03 ^A^ 1.26 ± 0.05 ^A^	8.76 ± 0.25 ^a^ 5.91 ± 0.17 ^a^ 1.10 ± 0.03 ^a^ 1.76 ± 0.05 ^a^	16.8 ± 0.3 ^B^ 8.40 ± 0.18 ^B^ 6.42 ± 0.20 ^B^ 1.90 ± 0.03 ^B^	19.2 ± 0.6 ^b^ 9.28 ± 0.13 ^b^ 8.02 ± 0.25 ^b^ 2.36 ± 0.02 ^b^	5.95 ± 0.10 ^A^ 2.90 ± 0.04 ^C^ 2.13 ± 0.03 ^C^ 0.86 ± 0.01 ^C^	8.43 ± 0.22 ^a^ 4.11 ± 0.10 ^c^ 3.02 ± 0.07 ^c^ 1.22 ± 0.03 ^c^	5.40 ± 0.30 ^A^ 2.54 ± 0.11 ^C^ 0.63 ± 0.03 ^A^ 2.18 ± 0.03 ^D^	7.91 ± 0.01 ^a^ 3.72 ± 0.00 ^c^ 0.92 ± 0.01 ^a^ 3.19 ± 0.01 ^d^

Note: Data expressed during the whole 30-day period studied as mean ± SEM. Statistical analysis was two-way ANOVA: In all cases, *p*-values both for diet and for sex were *p* < 0.0001, except for protein accumulation (*p* = 0.0003 for diet comparison), water content (*p* = 0.0049 for sex), lipid content (*p* = 0.0283 for sex), and protein content (not significant for sex). Bonferroni’s post hoc test of statistical significance, established at *p* < 0.05, is represented by different superscript letters.

**Table 3:** Nitrogen balance of rats fed a control, cafeteria, high-fat, or high-protein diet for 30 days.

	Standard Diet		Cafeteria Diet		High-Fat Diet		High-Protein Diet		ANOVA
	Female	Male	Female	Male	Female	Male	Female	Male	
Initial body N (g)	8.40 ± 0.17	12.7 ± 0.8	7.72 ± 0.13	12.7 ± 0.5	8.31 ± 0.11	12.3 ± 0.2	8.13 ± 0.41	13.0 ± 0.1	S
Final body N (g)	9.30 ± 0.25	15.6 ± 0.9	8.53 ± 0.20	15.1 ± 0.5	8.67 ± 0.19	13.3 ± 0.9	8.46 ± 0.30	15.1 ± 0.2	S
Ingested N (g)	13.5 ± 0.5	18.8 ± 1.0	20.3 ± 0.3	25.2 ± 0.2	9.22 ± 0.14	13.1 ± 0.3	23.3 ± 0.9	34.1 ± 0.1	D,S,I
Accrued N (g)	0.90 ± 0.14	2.84 ± 0.35	0.82 ± 0.18	2.37 ± 0.58	0.36 ± 0.11	1.03 ± 0.18	0.33 ± 0.13	2.15 ± 0.23	D,S
Accrued N (% of ingested)	6.58 ± 0.94	15.4 ± 1.8	4.08 ± 0.92	9.46 ± 2.0	3.89 ± 1.11	7.85 ± 1.10	1.37 ± 0.64	6.29 ± 0.58	D,S
Excreted N * (g)	12.6 ± 0.5	16.0 ± 1.0	19.5 ± 0.5	22.8 ± 0.7	8.86 ± 0.14	12.1 ± 0.3	23.0 ± 0.9	32.0 ± 0.2	D,S,I

Note: Data are expressed as mean ± SEM, and are represented as g of N in 30 days. * Excreted N was calculated as the difference between the ingested N and the accumulated N. Statistical analysis was two-way ANOVA. Only significant *p*-values are shown: Diet (D), sex (S), or their interaction (I).

**Table 4:** Plasma amino acid levels in rats fed a standard, cafeteria, high-fat, or high-protein diet for 30 days.

	Standard Diet		Cafeteria Diet		High-Fat Diet		High-Protein Diet		ANOVA
	Female	Male	Female	Male	Female	Male	Female	Male	
Ala	535 ± 53	517 ± 35	462 ± 28	575 ± 23	496 ± 37	560 ± 17	543 ± 37	589 ± 23	S
Ser	302 ± 33	248 ± 22	353 ± 39	403 ± 44	327 ± 15	340 ± 20	348 ± 18	351 ± 13	D
Thr	286 ± 42	242 ± 27	280 ± 42	296 ± 30	366 ± 31	255 ± 9	253 ± 23	291 ± 20	
Gly	210 ± 26	262 ± 25	271 ± 33	460 ± 55	262 ± 8	346 ± 25	408 ± 23	511 ± 19	D,S
Pro	279 ± 46	299 ± 6	240 ± 24	286 ± 8	247 ± 34	265 ± 16	410 ± 26	480 ± 46	D
Asp	21.0 ± 3.9	13.5 ± 1.2	13.2 ± 1.4	15.2 ± 0.8	17.0 ± 1.6	21.2 ± 1.6	15.1 ± 1.6	18.2 ± 1.3	I
Asn	63.3 ± 11.1	76.0 ± 6.3	59.2 ± 8.0	86.1 ± 11.0	58.3 ± 13.2	73.9 ± 6.6	79.6 ± 8.6	101 ± 7.7	S
Glu	82.1 ± 9.4	104 ± 10.8	89.7 ± 7.5	121 ± 7.4	98.7 ± 14.0	134 ± 7.5	90.6 ± 6.9	128 ± 10.2	S
Gln	682 ± 69	735 ± 79	535 ± 36	718 ± 38	792 ± 41	890 ± 30	632 ± 29	656 ± 23	D,I
Val	177 ± 25	164 ± 9	142 ± 19	166 ± 14	107 ± 21	116 ± 10	211 ± 11	258 ± 19	D
Leu	207 ± 24	189 ± 10	155 ± 14	186 ± 7	151 ± 17	156 ± 11	197 ± 10	242 ± 11	D
Ile	110 ± 10	102 ± 4	100 ± 11	113 ± 8	76.4 ± 9.3	84.0 ± 4.7	116 ± 6	144 ± 7	D
Arg	155 ± 21	162 ± 16	174 ± 17	202 ± 12	136 ± 23	152 ± 10	138 ± 11	158 ± 23	
Ornithine	49.5 ± 10.8	60.9 ± 5.7	38.9 ± 4.4	68.5 ± 6.5	74.6 ± 9.9	78.8 ± 8.8	87.6 ± 8.1	143 ± 24	D,S
Citrulline	104 ± 11	91.0 ± 9.3	87.3 ± 6.0	93.7 ± 3.7	92.1 ± 3.7	104 ± 4	94.6 ± 4.0	102 ± 10	
Met	73.9 ± 10.7	67.7 ± 5.6	57.9 ± 3.6	71.2 ± 4.5	61.4 ± 6.6	70.9 ± 2.9	66.0 ± 2.4	80.9 ± 6.6	
Phe	71.8 ± 6.2	68.9 ± 4.1	56.8 ± 3.4	70.4 ± 2.6	66.4 ± 7.3	79.8 ± 3.6	67.8 ± 3.7	81.6 ± 4.3	S
Tyr	55.9 ± 11.9	66.5 ± 6.6	42.5 ± 7.2	79.5 ± 3.2	60.7 ± 8.7	111 ± 5	60.7 ± 4.9	76.7 ± 5.9	D,S,I
His	69.5 ± 8.9	65.4 ± 5.3	55.8 ± 2.2	71.9 ± 5.4	53.3 ± 6.0	63.6 ± 2.9	55.7 ± 1.8	67.4 ± 1.4	S
Lys	382 ± 44	284 ± 19	346 ± 41	344 ± 36	293 ± 22	190 ± 10	461 ± 22	405 ± 17	D,S
Trp	127 ± 15	106 ± 16	98.8 ± 10.5	123 ± 7	126 ± 6	130 ± 10	139 ± 4	131 ± 8	

Note: The data (µM) correspond to the mean ± SEM of 6–8 different animals. Statistical analysis was two-way ANOVA: *p*-values are for diet (D), sex (S), or interaction (I).
